# Rational design of cyclopropane-based chiral PHOX ligands for intermolecular asymmetric Heck reaction

**DOI:** 10.3762/bjoc.10.158

**Published:** 2014-07-07

**Authors:** Marina Rubina, William M Sherrill, Alexey Yu Barkov, Michael Rubin

**Affiliations:** 1 Department of Chemistry, University of Kansas, 1251 Wescoe Hall Drive, Lawrence, KS 66045-7582, USA; 2 Department of Chemistry, Ural Federal University, pr. Lenina 51, 620000 Ekaterinburg, Russian Federation

**Keywords:** asymmetric catalysis, chiral phosphine ligands, cyclopropane, Heck reaction, organophosphorus, transition metal catalysis

## Abstract

A novel class of chiral phosphanyl-oxazoline (PHOX) ligands with a conformationally rigid cyclopropyl backbone was synthesized and tested in the intermolecular asymmetric Heck reaction. Mechanistic modelling and crystallographic studies were used to predict the optimal ligand structure and helped to design a very efficient and highly selective catalytic system. Employment of the optimized ligands in the asymmetric arylation of cyclic olefins allowed for achieving high enantioselectivities and significantly suppressing product isomerization. Factors affecting the selectivity and the rate of the isomerization were identified. It was shown that the nature of this isomerization is different from that demonstrated previously using chiral diphosphine ligands.

## Introduction

The asymmetric Heck reaction is one of the most powerful and versatile processes for the enantioselective construction of new carbon–carbon bonds. Intramolecular versions of this reaction catalysed by palladium complexes with BINAP and related diphosphine ligands [1–2] allow for efficient installation of tertiary and quaternary chiral centres leading to a rapid increase of molecular complexity [3–5]. To date, various modes of this transformation are being successfully employed in the synthesis of complex organic molecules [6–14].

**Scheme 1:**
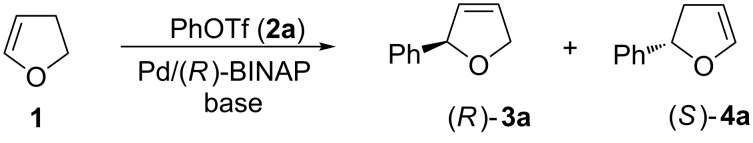
Intermolecular asymmetric Heck reaction by Hayashi [16].

**Scheme 2:**
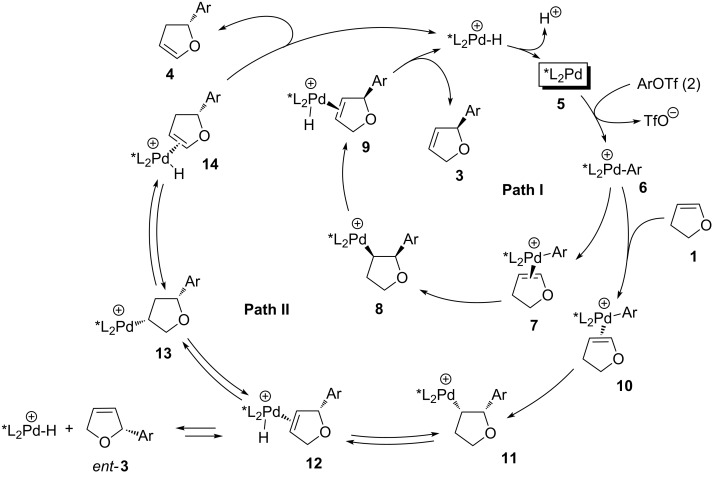
Mechanistic rationale of asymmetric Heck reaction.

Considerable achievements have also been made towards the application of BINAP-type ligands in the intermolecular asymmetric Heck reaction [15]. This reaction was pioneered by Hayashi [16], who demonstrated the arylation of dihydrofuran (**1**) with phenyl triflate (**2a**) (Scheme 1) in the presence of (*R*)-BINAP [16–18] produced isomeric dihydrofurans **3a** and **4a**, with the latter being the major product, due to substantial isomerization of the double bond. Depending on the reaction conditions, moderate to good selectivities toward formation of **4a** were observed. Remarkably, the obtained products, “normal” **3a** and “isomerized” **4a**, had the opposite absolute configurations of the stereogenic center at C2. Moreover, it was found that the enantioselectivity improved during the reaction course. The mechanistic rationale proposed by Hayashi [16] fully accounts for the observed stereoselectivity change (Scheme 2). The catalytic cycle begins with the oxidative addition of Pd(0) species **5** into the aryl triflate **2** resulting in the formation of cationic complex **6**. The latter can coordinate to either of the prochiral faces of dihydrofuran (**1**) affording diastereomeric η^2^-complexes **7** and **10**. Subsequent carbopalladation, followed by β-hydride elimination, produces species **9** and **12**, respectively. It was proposed that the diastereomeric complex **12** has a higher propensity toward further hydropalladation than **9**. Accordingly, the latter species releases the (*S*)-enantiomer of 2,5-dihydrofuran **3** (path I), while the former undergoes a series of reversible hydropalladations and β-hydride eliminations, resulting in the formation of a thermodynamically more favoured η^2^-complex **14**, which ultimately produces the (*R*)-enantiomer of the isomeric product **4**.

**Figure 1:**
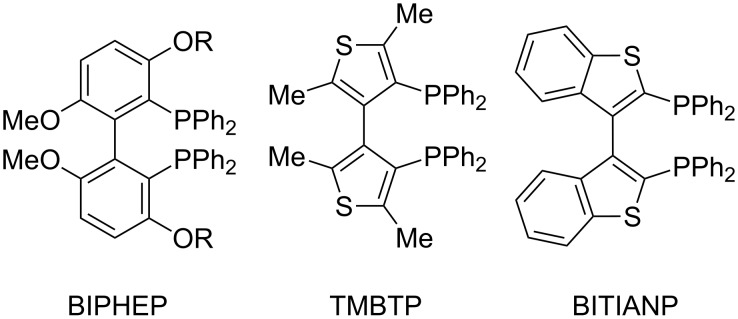
Chiral diphosphine ligands used for intermolecular asymmetric Heck reaction.

Later, a number of research groups pursued the design of alternative diphosphine ligands to achieve better regio- and enantioselectivity in the intramolecular Heck reaction. Several derivatives of BINAP [19–20] and other chiral diphosphines [21–27] including TMBTP [28–31], BIPHEP [32–34], BITIANP [30,35] (Figure 1) were tested, some of which provided improved selectivity. Nevertheless, in all cases predominant or exclusive formation of the isomerized product **4** was observed.

**Figure 2:**
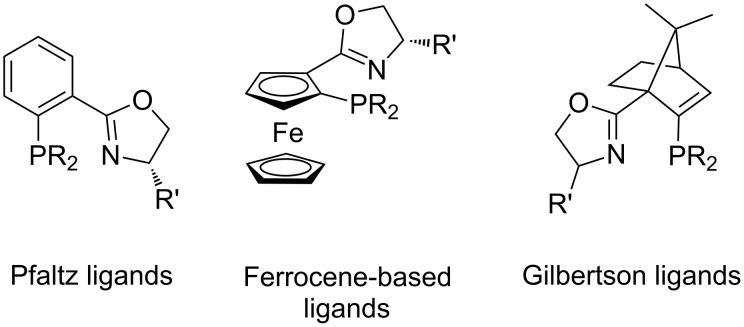
Chiral phosphanyl-oxazoline (PHOX) ligands used for intermolecular asymmetric Heck reaction.

At the same time, several mixed hetereoatom ligands of the P–S [36–37], P–O [38], and N–N [39–40] type have also been explored in the intermolecular Heck arylation; however, they demonstrated in most cases only marginal regio- and enantioselectivities. On the other hand, superior results were obtained using chiral ligands of the P,N-type [15,41–44]. Particularly, excellent enantioselectivities were achieved using different variations of phosphanyl-oxazoline (PHOX) ligands [45–52], originally introduced by Pfaltz (Figure 2) [53–54]. The remarkable, yet not fully understood feature of PHOX ligands is their low tendency to promote C=C-bond isomerization [45–52]. Thus, in contrast to the diphosphines, PHOX ligands produced dihydrofuran **3** with very high selectivity. Structural modification of the flat ortho-phenylene tether in the Pfaltz ligand through the incorporation of additional chirality elements into the ligand backbone allowed for significant improvement of the enantioselectivity. Thus, ferrocene-based ligands introduced by Dai and Hou [55–56], and Guiry [57–58] (Figure 2) were employed in the asymmetric Heck reaction of different cyclic olefins. Furthermore, Gilbertson demonstrated PHOX ligands featuring apobornene backbone (Figure 2) exhibit outstanding activities and selectivities in the arylation and alkenylation of different cyclic substrates [59]. A highly efficient asymmetric arylation in the presence of sugar-derived phosphite-oxazoline ligands was reported by Diéguez and Pàmies [47–48].

PHOX ligands are very appealing due to their high catalytic potential and modular design, which permits easy preparation of a series of analogues via the same synthetic route. To date, however, general approach to the ligand design has been largely empirical due to a poor understanding of the factors affecting the activity of the corresponding catalytic systems and the operating modes of asymmetric induction imparted by the employed chiral ligands. In our investigation, we decided to benefit from a well-established strategy commonly used in medicinal chemistry. According to this approach conformationally constrained cyclic analogues of biologically active molecules are employed for elucidation of important mechanisms and identifying critical enzyme binding sites. Analogously, we anticipated that incorporation of a three-membered cycle in the ligand structure [60–63] would impart rigidity to the ligand backbone and provide conformationally constrained systems with amplified steric effects, which can be easily modelled and predicted. This, in turn, could be used to rationally design the ligand structure en route to more efficient catalytic systems. In 2008 we communicated the design and synthesis of a novel series of PHOX ligands featuring a chiral cyclopropyl backbone, as well as their employment in the enantioselective intermolecular Heck arylation reaction [64]. Herein we describe the full account on this investigation, including the results of the structure–activity studies and provide our insight into the origins of the enantioselectivity of this transformation and factors controlling the rate of isomerization reaction.

## Results and Discussion

**Scheme 3:**
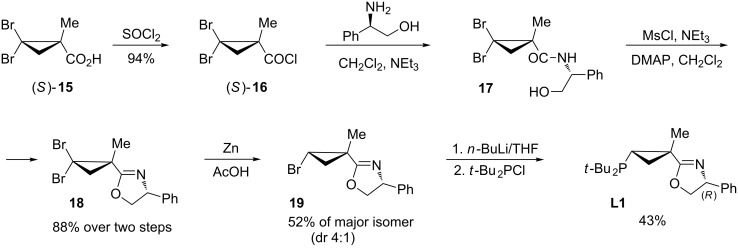
Synthetic scheme for preparation of PHOX ligands with chiral cyclopropyl backbone.

Our approach to the PHOX ligands with a chiral cyclopropyl backbone is presented in Scheme 3. The synthesis began from optically active 1-methyl-2,2-dibromocyclopropanecarboxylic acid (**15**) [65] readily available in both enantiomeric forms. The *S*-enantiomer of acid **15** was converted into acyl chloride (*S*)-**16**. Subsequent acylation of (*R*)-phenylglycinol with (*S*)-**16** afforded amide **17**, which was subjected to cyclization in the presence of mesyl chloride and a base providing dihydrooxazole **18**. Diastereoselective partial reduction of the dibromocyclopropane moiety with zinc dust in glacial acetic acid produced a 1:4 mixture of *trans*- and *cis*-bromocyclopropanes **19**, which were separated by column chromatography. Lithium to halogen exchange followed by trapping of the resulting cyclopropyllithium species with chlorophosphine produced ligand **L1** (Scheme 3).

Ligand **L1** once obtained, was tested in the asymmetric arylation reaction of 2,3-dihydrofuran under various reaction conditions (Table 1). It was found that the reaction proceeded efficiently, yet with only moderate enantioselectivity, in the presence of palladium acetate and Hünig’s base (Table 1, entry 3). Interestingly, the employment of proton sponge as a base resulted in significant isomerization of product **3a** into the more thermodynamically stable dihydrofurans **4a** and **20a**. Close monitoring of the reaction by chiral GC revealed, that the initially formation of “normal” product **3a** is observed (Table 1, entry 4); however, by the time when starting material **1** was completely consumed, the entire amount of **3a** produced was transformed into **4a** (Table 1, entry 5). Remarkably, the absolute configuration at C2 did not change at all through the reaction course; moreover, the optical purity of both products **3a** and **4a** remained constant (Table 1, entries 4 and 5). This feature makes this isomerization mechanistically distinct from the one reported by Hayashi (vide supra).

**Table 1:** Selected results on optimization of the reaction conditions for asymmetric Heck arylation using **L1**.

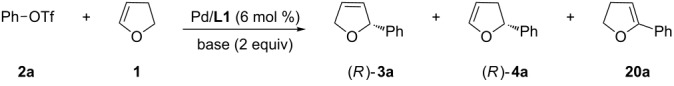							

Entry	Pd cat.	Base	Solvent	Time/Temp	**3a**:**4a**	ee, %^a^	conv, %^b^

1	Pd_2_dba_3_·CHCl_3_	EtN(iPr)_2_	benzene	3 d/70 °C	19:1	90	15
2	Pd_2_dba_3_·CHCl_3_	EtN(iPr)_2_	THF	20 h/85 °C	10:1	85	60
3	Pd(OAc)_2_	EtN(iPr)_2_	THF	20 h/85 °C	11:1	83	99
4	Pd(OAc)_2_	proton sponge	THF	20 h/60 °C	10:1	88	45
5	Pd(OAc)_2_	proton sponge	THF	70 h/60 °C	>1:50^c^	85	99
6	Pd(OAc)_2_	proton sponge	THF	20 h/90 °C	>1:50^c^	82	99

^a^Ee's of major regioisomers are listed. ^b^Conversion by GC. ^c^Formation of small amounts of dihydrofuran **20a** was observed.

**Figure 3:**
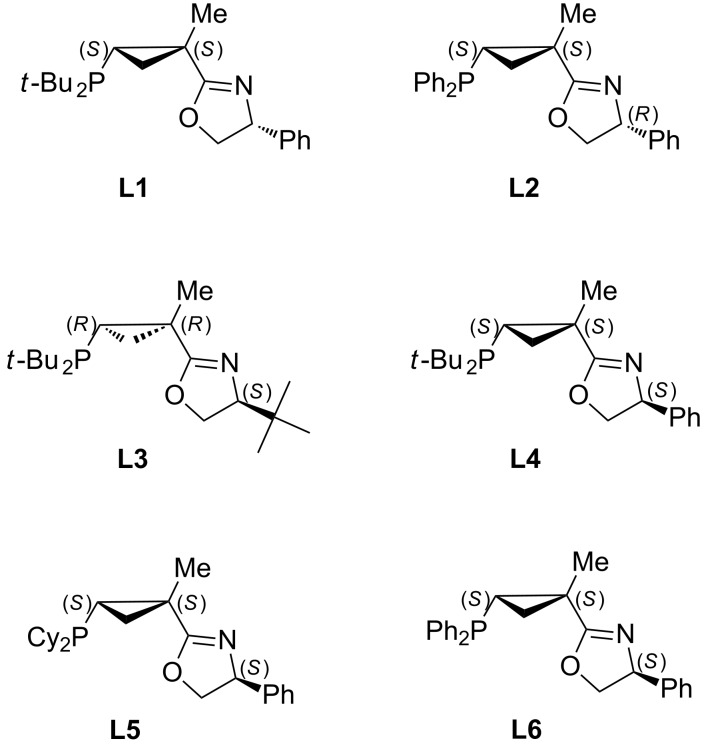
PHOX ligands with chiral cyclopropyl backbone employed in this study.

To better understand the factors affecting the selectivity and efficiency of the asymmetric arylation, we have prepared two more analogues of **L1**: ligand **L2**, possessing a diphenylphosphanyl group and ligand **L3** derived from *tert*-leucinol (Figure 3). Not surprisingly, installation of the less hindered phosphorus moiety in **L2** negatively affected the asymmetric induction: the corresponding product **3a** was obtained in only 78–79% ee (Table 2, entries 3 and 4). However, in contrast to **L1** (Table 2, entries 1 and 2) the selectivity toward **3a** in the reaction using **L2** remained high, regardless of the base used.

**Table 2:** Screening of **L1**–**L3** in the asymmetric Heck arylation of dihydrofuran **1**.

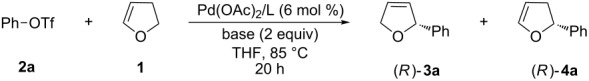					

Entry	Ligand	Base	**3a**:**4a**	ee, %^a^	conv, %^b^

1	**L1**^c^	EtN(iPr)_2_	11:1	83	99
2	**L1**^c^	proton sponge	>1:50	82	99
3	**L2**	EtN(iPr)_2_	20:1	79	99
4	**L2**	proton sponge	15:1	78	99
5	**L3**	EtN(iPr)_2_	7:1	87	35
6	**L3**	proton sponge	1.4:1	84^d^	80

^a^Enantioselectivity of a major product. ^b^Conversions by GC. ^c^Results from Table 1. ^d^Enantioselectivity of product (*R*)-**4a** was 80%.

Modification of the dihydrooxazole moiety by installation of a bulky *tert*-butyl group was pursued in attempt to improve the enantioinduction of our catalytic system. Indeed, a number of previously reported PHOX ligands derived from *tert*-leucinol were shown to provide superior enantioselectivities compared to their analogues obtained from less bulky amino alcohols [54,57,59]. However, the arylation carried out in the presence of **L3** proceeded much more sluggishly (Table 2, entries 5 and 6), and allowed for only insignificant improvement in enantioselectivity (84–87% ee). Most remarkably, the same (*R*)-enantiomer of product **3** was obtained, despite the opposite absolute configuration of **L3** with respect to **L1** (Figure 3). In other words, switching from Ph to *t*-Bu substituent in the dihydrooxazole ring of the ligand resulted in a reversal of enantioselectivity.

**Scheme 4:**
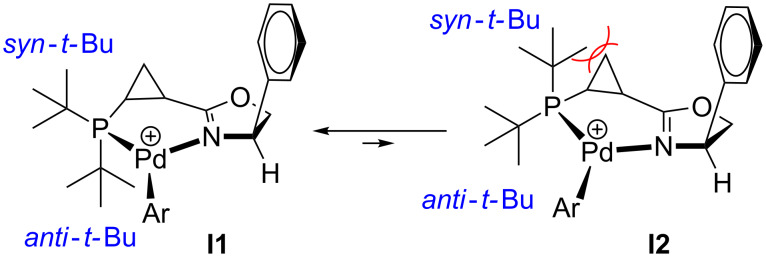
Conformational equilibrium in cationic arylpalladium(II) complexes with chiral ligand **L1**.

**Figure 4:**
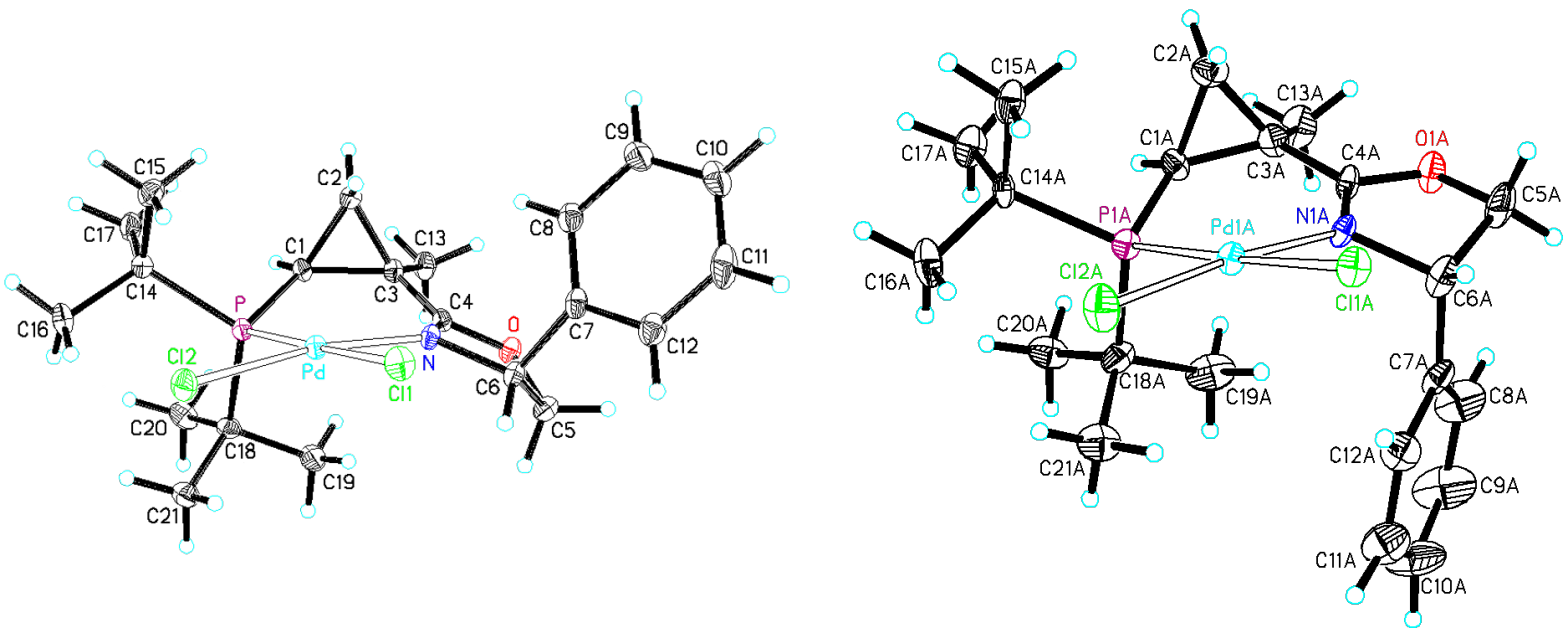
X-ray structures of complexes (**L1**)PdCl_2_ (left) and (**L4**)PdCl_2_ (right). These structures were originally communicated in [64].

**Scheme 5:**
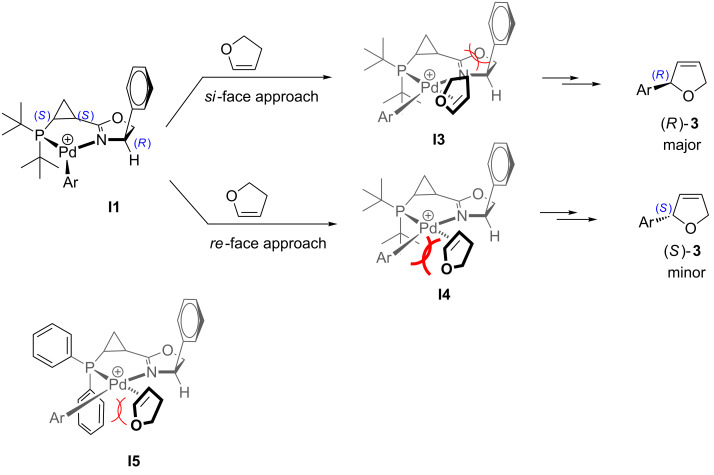
For discussion on asymmetric induction imparted by chiral ligands **L1** and **L2** (originally published in [64]).

Such an unexpected change in the catalyst selectivity motivated us to perform structural analysis of the key intermediate complexes invoked in the catalytic cycle of the Heck arylation. First, we assessed the possibility of conformational equilibrium for the six-membered arylpalladium species bearing **L1** (Scheme 4). The non-planar six-membered palladacycle [66–69] can potentially adopt one of two conformations: **I1**, in which the *syn*-*tert*-butyl substituent at phosphorus assumes a pseudo-equatorial position, whereas the *anti*-*tert*-butyl substituent is pseudo-axial; and **I2**, where this relationship is reversed (Scheme 4). Analysis of these two conformations suggests that steric repulsions between the axial *syn*-substituent and the methylene group in cyclopropane makes conformation **I2** thermodynamically disfavored compared to **I1**. This hypothesis was also supported by a single crystal X-ray analysis of (**L1**)PdCl_2_ complex (Figure 4). The resolved crystal structure clearly shows that the *syn*-(C14) and *anti*-substituent (C18) at phosphorus adopt a pseudo-equatorial and a pseudo-axial position, respectively. It would be reasonable to assume that the strained and rigid cyclopropyl backbone renders the six-membered palladacycle particularly inflexible, thus significantly suppressing conformational fluctuations throughout the catalytic cycle. Furthermore, coordination of the soft π-ligand dihydrofuran should take place predominantly *trans* to a soft phosphorus atom [70–72] (Scheme 5). In this case, the *re*-face approach (**I4**) is encumbered by a large pseudo-axial *tert*-butyl group, while the *si*-face approach (**I3**) is also somewhat hindered by a pseudo-axial *syn*-phenyl substituent in dihydrooxazole ring. As a result, the (*R*)-enantiomer of the product was predominantly formed, albeit with moderate enantioselectivity. Analogously, in the intermediate **I5** derived from chiral ligand **L2**, the less bulky pseudo-axial phenyl substituent at phosphorus blocks the *re*-face approach even less efficiently, which ultimately results in a further decrease of enantioselectivity (Scheme 5).

**Scheme 6:**
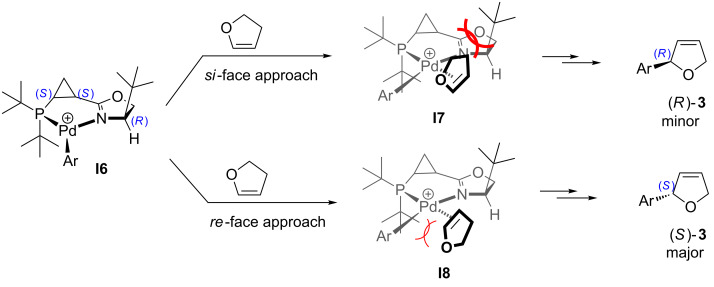
For discussion on asymmetric induction imparted by chiral ligands **L3** (originally published in [64]).

The reversal of enantioselectivity observed in the reaction carried out in the presence of **L3** was explained in a similar fashion (Table 2, entries 5 and 6, Scheme 6). Thus, a bulky *tert*-butyl group in the dihydrooxazole ring creates the increased steric hindrance, which does not allow for the *si*-face approach resulting in the reaction proceeding predominantly from the *re*-face, providing the (*S*)-enantiomer of **3** (Scheme 6). The fact that in both intermediates **I7** and **I8** dihydrofuran experiences certain impediment on approach to palladium may also be responsible for the observed decrease in the reaction rate.

**Scheme 7:**
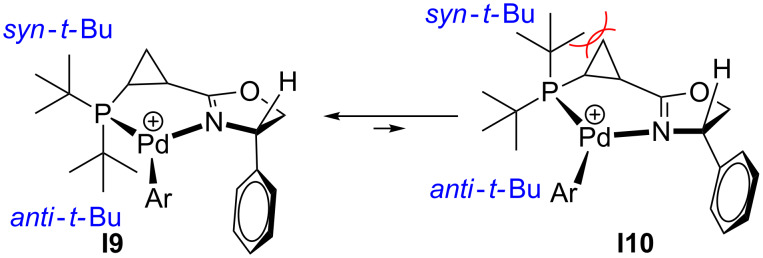
Conformational equilibrium in cationic arylpalladium(II) complexes with chiral ligand **L4**.

**Scheme 8:**
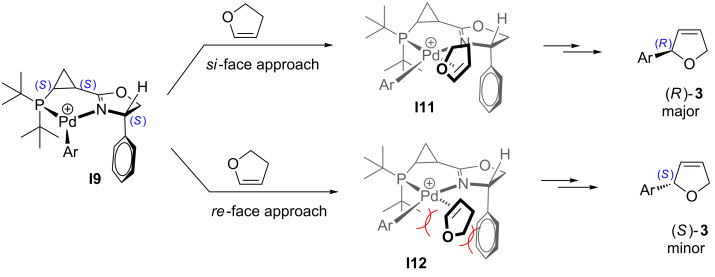
For discussion on asymmetric induction imparted by chiral ligands **L4** (originally published in [64]).

Based on this analysis, we rationalized that the “wrong” relative configuration of the stereogenic centers in ligands **L1**, **L2** and **L3** could be responsible for the observed marginal enantioselectivity of the corresponding catalytic systems. We envisioned that inverting the absolute configuration of the asymmetric center at C4 in the dihydrooxazole ring might potentially help to improve the enantioselectivity of the arylation reaction. Indeed, it is reasonable to propose that the inversion of the stereogenic center in the dihydrooxazole ring should not significantly affect the thermodynamic equilibrium of the corresponding palladacycle conformations **I9** and **I10** (Scheme 7), as compared to **I1** and **I2** (Scheme 4). Thus, the cationic palladacycle with (*S*,*S*,*S*)-ligand **L4** would still predominantly adopt conformation **I9** to avoid the unfavorable steric interaction between the pseudo-axial *syn*-*tert*-butyl group and the methylene group of the cyclopropane (Scheme 7). Accordingly, a synergistic steric effect of both the axial P–*t-*Bu group and a bulky substituent at C4 in dihydrooxazolyl moiety observed in the alternative (*S*,*S*,*S*)-configuration of the ligand would now provide efficient blocking of the both bottom quadrants thereby completely averting the *re*-face attack (**I12**, Scheme 8). On the other hand, the *si*-face attack should become more favorable after the removal of a bulky group obstructing the top right quadrant (**I11**, Scheme 8 vs **I3**, Scheme 5). Ultimately, if the above assumptions are correct, this change should result in enhanced enantioselectivity of the asymmetric arylation in the presence of ligand **L4** in favor of the (*R*)-enantiomer of the product **3**.

With this idea in mind, we prepared a new series of ligands with the (*S,S,S*)-absolute configuration using the synthetic approach described above (Scheme 3), starting from acid chloride (*S*)-**16** and (*S*)-phenylglycinol. Additional diversification of the ligand structure was achieved by varying the chlorophosphine source. Thus, employment of di-*tert*-butylchlorophosphine, chlorodicyclohexylphosphine, and chlorodiphenylphosphine at the last step of the sequence provided ligands **L4, L5**, and **L6**, respectively (Figure 3). Crystallographic data obtained for the (**L4**)PdCl_2_ complex (Figure 4) completely confirmed the preference of conformation **I9** vs **I10** (Scheme 7). It should be pointed out, that the resolved crystal structure of (**L4**)PdCl_2_ complex shows four sets of crystallographically independent molecules. However, all of them have nearly identical palladacycle conformations with the molecule shown in Figure 4 [64]. An overlay of X-ray structures obtained for (**L1**)PdCl_2_ and (**L4**)PdCl_2_ complexes demonstrated that all atoms of the palladacycle, cyclopropyl ring, and both *tert*-butyl substituents can be almost perfectly superimposed, which for both ligand configurations, confirms the strong preference of a conformation in which the *syn*-*tert*-Bu substituent (C14) and the *anti*-*tert*-Bu substituent (C18) at phosphorus assume pseudo-equatorial and pseudo-axial positions, respectively. Remarkably, X-ray analysis has also demonstrated that the phenyl substituent at C4 of dihydrooxazole ring adopts a pseudo-axial position thereby completely blocking any potential re-face attack (Scheme 8).

Ligands **L4**, **L5**, and **L6** once obtained were tested in the asymmetric arylation of dihydrofuran **1** (Table 3). Gratifyingly, right along with our expectations, the entire series of (*S*,*S*,*S*)-ligands **L4**–**L6** not only provided a significant improvement in enantioselectivity, but also helped to suppress the unwanted isomerization of **3** into **4**, as compared to the diastereomeric ligand series (**L1**–**L3**, Table 2). Remarkably, changing the absolute configuration of the stereocenter in the dihydrooxazole ring did not cause the change of the absolute configuration of the product. This is in contrast to the reactions performed using most known PHOX ligands, in which configuration of the oxazoline moiety usually determines the stereochemical outcome of the reaction (however, in the reactions using PHOX ligands bearing a very bulky planar or axially chiral backbone, the enantiomeric outcome is controlled by the absolute configuration of the backbone rather than that of the oxazoline ring; for discussion, see [15]). Thus, employment of **L4** and **L5** afforded dihydrofuran (*R*)-**3** with very high enantioselectivity regardless of the base used (Table 3, entries 1–6); however, the reactions proceeded more sluggishly in the presence of Hünig’s base (Table 3, entries 2 and 5). Employment of proton sponge helped boost the reaction rate in the arylation catalyzed by both **L4** and **L5** (Table 3, entries 3 and 6). Yet, significant isomerization of **3** into **4** was observed with this base when the reaction catalyzed by Pd/**L4** complex was allowed to run for an additional 20 h (Table 3, note c). Employment of the diphenylphosphanyl ligand **L6** provided lower enantioselectivity (Table 3, entries 7 and 8), which can be attributed to decreased steric demands created by phenyl groups at phosphorus as compared to the *tert*-butyl (**L4**) and cyclohexyl (**L5**) substituents.

**Table 3:** Screening of **L4**–**L6** in the asymmetric Heck arylation reaction.

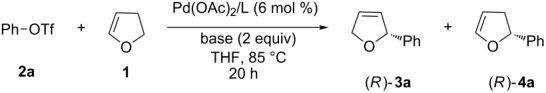					

Entry	Ligand	Base	**3a**:**4a**	ee (**3a**), %	Conv, %^a^

1	**L4**	EtN(iPr)_2_	>50:1	98	53
2	**L4**	EtN(iPr)_2_	16:1	98	97^b^
3	**L4**	proton sponge	>50:1^c^	98	74
4	**L5**	EtN(iPr)_2_	>50:1	94	71
5	**L5**	EtN(iPr)_2_	40:1	94	90^b^
6	**L5**	proton sponge	29:1	95	99
7	**L6**	EtN(iPr)_2_	16:1	88	76
8	**L6**	proton sponge	>50:1	86	83

^a^Conversions by GC. ^b^Conversion after 2 days at 85 °C. ^c^When the reaction was allowed to stir for an additional 20 h, the product ratio changed to 2:1. The enantioselectivities of products (*R*)-**3a** and (*R*)-**4a** in this case were found to be 98% and 97%, respectively.

**Scheme 9:**
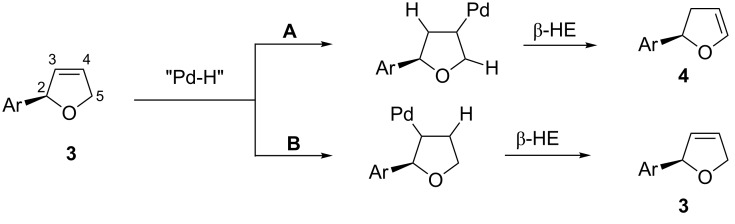
Mechanism of migration of C=C double bond leading to isomerization of product **3** into product **4**.

**Scheme 10:**
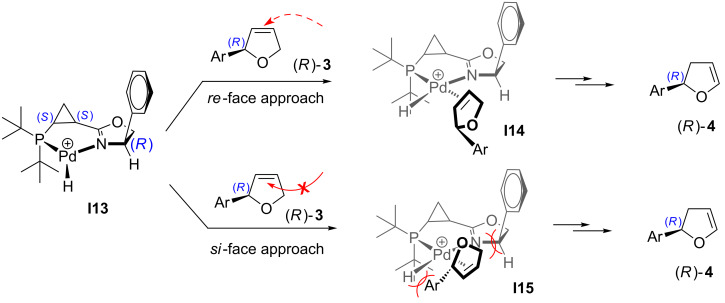
For discussion on isomerization **3**→**4** imparted by Pd/**L1** complex (originally published in [64]).

The different tendencies of Pd/**L1** and Pd/**L4** catalyst systems to promote isomerization of product **3** into **4** can be rationalized as follows. As discussed above (Scheme 2), the isomerization process involves reversible hydropalladation of the double bond of product **3**. The migration of the double bond can be realized only when hydropalladation of **3** occurs with addition of palladium to C4 (Scheme 9, path A), whereas the opposite regioselectivity of hydropalladation would ultimately lead, after the subsequent β-hydride elimination, back to compound **3** (Scheme 9, path B). The diastereoselectivity of the hydropalladation of **3** by Pd/**L1** hydride species **I13** is controlled as shown in Scheme 10. Thus, it seems impossible to realize the *si-*face approach of palladium hydride species **I13** to the double bond of **3** due to severe steric hindrance between the di(*tert*-butyl)phospanyl group of the ligand and the aryl substituent in **3** on one side, and between the phenyl substituent in dihydrooxazole ring and C5-methylene of dihydrofuran **3** on the other (**I15**, Scheme 10). However, the absence of any significant steric interference upon alternative *re*-face approach makes this alternative mechanistic channel available for isomerization (**I14**, Scheme 10).

**Scheme 11:**
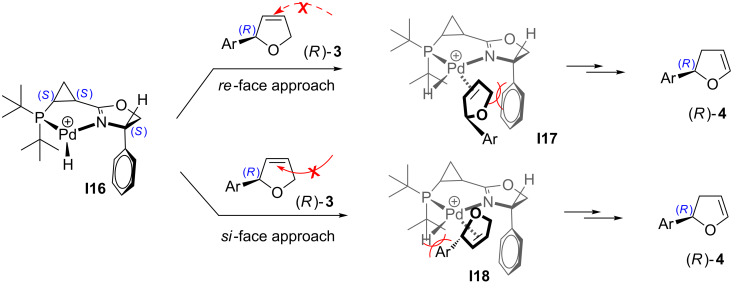
For discussion on isomerization **3**→**4** imparted by Pd/**L4** complex (originally published in [64]).

Two potential pathways for hydropalladation of **3** by the diastereomeric Pd/**L4** hydride species **I16** are shown in Scheme 11. In conjunction with **L1**-derived complex **I15** (Scheme 10), complex **I18** produced via the *si*-face approach should be highly disfavored (Scheme 11). In this case, however, an alternative complex **I17** resulting from the *re*-face attack should also experience steric repulsion between the C5-methylene of dihydrofuran **3** and a pseudo-equatorial phenyl substituent in dihydrooxazole ring (Scheme 11). Accordingly, complex **I17** should be much more unfavorable compared to **L1**-derived complex **I14**, where such interaction does not occur (Scheme 10). As a result, both mechanistic channels for isomerization of compound **3** into **4** should be suppressed in this case. It should be mentioned, however, that electronic density at the phosphine moiety of the ligand also notably affects the propensity of the corresponding catalyst to promote the isomerization. Thus, our experiments indicate that in the series of di(*tert*-butyl)-, dicyclohexyl-, and diphenylphosphanyl-containing ligands (**L4**→**L6**), the former has the highest tendency to induce isomerization while the latter has the lowest (Table 3). A similar electronic effect was previously observed in the asymmetric Heck arylation in the presence of diphosphine-oxazoline ferrocenyl ligands [56].

Next, the most efficient ligands **L4** and **L5** were tested in the asymmetric arylation of dihydrofuran **1** against various aryl triflates (Table 4). It was found that all reactions catalyzed by Pd/**L4** provided excellent enantioselectivities (98–99%) regardless of the nature of the aryl triflate (Table 4, entries 1–5). However, the reactions carried out in the presence of **L4**/Hünig’s base combination proceeded much more sluggishly; as a result, the selectivity toward formation of **3** was slightly lower in these cases. Reactions performed in the presence of Pd/**L5** catalyst and proton sponge proceeded much faster, albeit providing somewhat lower ee's (Table 4, entries 6–10). In contrast to the Pd/**L4**-catalyzed reactions, enantioselectivities in this case varied slightly depending on the aryl triflate used, with the highest value obtained from 1-naphthyl triflate (96%, Table 4, entry 9) and the lowest from 2-naphthyl triflate (87%, Table 4, entry 10). Interestingly, the electronic nature of the aryl triflate had a pronounced effect on the reaction rate, which is best seen in the Pd/**L5** series of catalyzed reactions. Thus, electron-rich aryl triflates (Table 4, entries 6, 7, and 9) reacted much faster than the electron-poor analog **2d** (Table 4, entry 8). Furthermore, a remarkable difference between the reactivity of 1- and 2-naphthyl triflates was also observed, suggesting the reaction is also sensitive to sterics (Table 4, entries 9 and 10).

**Table 4:** Asymmetric arylation of dihydrofuran with aryl tiflates.

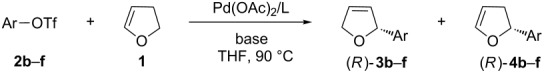							

Entry	Aryl		Ligand/Base	Time, h	**3**:**4**	ee (**3**), %	Conv, %^a^

1	*p*-Me-C_6_H_4_	**2b**	**L4**/Hünig’s base	48	16:1	99	96
2	*p*-MeO-C_6_H_4_	**2c**	**L4**/Hünig’s base	20	17:1	98	98
3	*p*-CF_3_-C_6_H_4_	**2d**	**L4**/Hünig’s base	48	>50:1	98	58
4	1-Nphth	**2e**	**L4**/Hünig’s base	48	18:1	98	70^b^
5	2-Nphth	**2f**	**L4**/Hünig’s base	20	>50:1	98	32^b^
6	*p*-Me-C_6_H_4_	**2b**	**L5**/proton sponge	6	39:1	95	93
7	*p*-MeO-C_6_H_4_	**2c**	**L5**/proton sponge	6	35:1	92	99
8	*p*-CF_3_-C_6_H_4_	**2d**	**L5**/proton sponge	20	42:1	91	95
9	1-Nphth	**2e**	**L5**/proton sponge	6	31:1	96	94^b^
10	2-Nphth	**2f**	**L5**/proton sponge	20	17:1	87	100^c^

^a^Conversion by GC. ^b^Formation of ca.10% of naphthalene was observed. ^c^Formation of ca. 20% of naphthalene was observed.

We also tested all new ligands **L1**–**L6** in the asymmetric Heck arylation of cyclopentene (Table 5). Initial experiments conducted under the conditions optimized for arylation of dihydrofuran **1** provided no reaction with cyclopentene **21**. Additional optimization revealed that reasonable reaction rates can be achieved only in the presence of Pd(dba)_2_ catalyst and proton sponge. It should be mentioned that employment of Pd_2_(dba)_3_·CHCl_3_ catalyst in place of Pd(dba)_2_ provided no reaction. Generally, the enantioselectivities obtained in this transformation (Table 5) were somewhat lower than those obtained in the arylation of dihydrofuran (Table 2 and Table 3) for all ligands tested except **L4**. Notably, similarly to the arylation of dihydrofuran (Table 2 and Table 3), the isomerization rates (**22**→**23**) in this transformation were significantly lower in the reactions carried out in the presence of ligands with the (*S*,*S*,*S*) absolute configuration (**L4**–**L6**, Table 5, entries 4–6), as compared to the ligands in the diastereomeric series (**L1**–**L3**, Table 5, entries 1–3).

**Table 5:** Evaluation of Ligands **L1**–**L6** in the intermolecular asymmetric Heck reaction of phenyl triflate (**2a**) with cyclopentene (**19**).

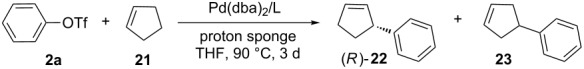					

Entry	Ligand	**22**:**23**	ee (**22**), %	Conv, %^a^	Yield, %*^b^*

1	**L1**	12:1	81	99	85
2	**L2**	15:1	86	95	80
3	**L3**	13:1	82	15	ND
4	**L4**	27:1	92	32	ND
5	**L5**	44:1	89	96	80
6	**L6**	40:1	80	60	ND

^a^Conversion by GC. ^b^Isolated yields, obtained by standard aqueous work-up of the reaction mixture, followed by fractionation.

## Conclusion

In conclusion, a series of novel PHOX ligands featuring a chiral cyclopropyl backbone have been synthesized and examined in the intermolecular asymmetric Heck arylation of cyclic olefins. By lowering degrees of freedom in the catalyst structure through the introduction of additional conformation constrains, we have created a model catalytic system with predictable, tuneable and easily adjustable properties. Structure–activity relationship studies allowed for identifying the key topological and stereochemical features of the ligands, responsible for achieving high enantioselectivity and for suppressing product isomerization. This has resulted in the development of efficient catalytic systems demonstrating excellent enantioselectivities in the asymmetric arylation of dihydrofuran with various aryl triflates. It was also shown that the product isomerization in the presence of these ligands has a different nature from that reported previously using chiral diphosphine ligands. Furthermore, a number of factors were shown to affect the isomerization rate including the absolute configuration of the ligand, its electronic properties, and the base employed.

## Supporting Information

File 1 Detailed experimental procedures of chiral ligands **L2**, **L5**, and **L6**.
