# Supplementary material for: Rational design of cyclopropane-based chiral PHOX ligands for intermolecular asymmetric Heck reaction
Source: Beilstein J Org Chem. 2014 Jul 7;10:1536–48. doi: 10.3762/bjoc.10.158 (PMC4143097; doi:10.3762/bjoc.10.158)
Supplement: File 1 — Detailed experimental procedures of chiral ligands L2, L5, and L6. [file Beilstein_J_Org_Chem-10-1536-s001.pdf]

Supporting Information

for

Rational design of cyclopropane-based chiral PHOX ligands for intermolecular asymmetric Heck reaction

Marina Rubina[1], William M. Sherrill[1], Alexey Yu. Barkov[2] and Michael Rubin*[1]

Address: [1]Department of Chemistry, University of Kansas, 1251 Wescoe Hall Drive, Lawrence, KS 66045-7582, USA and [2]Department of Chemistry, Ural Federal University, pr. Lenina 51, 620000 Ekaterinburg, Russian Federation

Email: Michael Rubin - mrubin@ku.edu

*Corresponding author

Detailed experimental procedures of chiral ligands L2, L5, and L6

Table of Contents:

General Information S2
Synthesis of chiral phosphine ligands S3
Spectral charts S5

General Information

NMR spectra were recorded on a Bruker Avance DPX-400 instrument, equipped with a quadruple-band gradient probe (H/C/P/F QNP) or a Bruker Avance DRX-500 with a dual carbon/proton cryoprobe (CPDUL). ^{13}C and ^{31}P NMR spectra were registered with broad-band decoupling. The (+) and (−) designations represent positive and negative intensities of signals in ^{13}C DEPT-135 experiments.

GC–MS analyses were performed on a Shimadzu GC-2010 gas chromatograph interfaced to a Shimadzu GCMS 2010S mass selective detector, and equipped with an AOC-20i auto-injector and an AOC-20S auto-sampler tray (150 vials). 30 m × 0.25 mm × 0.25 μm capillary column, SHR5XLB, polydimethylsiloxane, 5% Ph was employed. Helium (99.96%), additionally purified by passing consecutively through a CRS oxygen/moisture/hydrocarbon trap (#202839) and VICI oxygen/moisture trap (P100-1), was used as a carrier gas. The same model of gas chromatograph, equipped with the same auto-injector, FID detector, and J&W CyclosilB column (30 m × 0.25 mm × 0.25 μm) or J&W CyclodexB column (30 m × 0.25 mm × 0.25 μm) was employed for chiral GC analyses. Hydrogen gas was used as both carrier gas and FID fuel; zero-grade air and zero-grade nitrogen were used as an oxidant and make-up gas, respectively, for the FID. All these gases were purified by passing through CRS #202839 traps.

Glassware employed in moisture-free syntheses was flame-dried in vacuum prior to use. Water was purified by dual stage deionization, followed by dual stage reverse osmosis. Anhydrous hexane, dichloromethane, and tetrahydrofuran were obtained by passing degassed HPLC-grade commercially available solvents consecutively through two columns filled with activated alumina (Innovative Technology). Anhydrous triethylamine was obtained by distillation of ACS-grade commercially available materials over calcium hydride in a nitrogen atmosphere. Glacial acetic acid was purchased form Acros Organics and used as received. Palladium complexes were obtained from Strem Chemicals. Preparations of starting materials, (4*R*)-2-[(1*S*,2*S*)-2-bromo-1-methylcyclopropyl]-4-phenyl-4,5-dihydro-1,3-oxazole (**19**) and (4*S*)-2-[(1*S*,2*S*)-2-bromo-1-methylcyclopropyl]-4-phenyl-4,5-dihydro-1,3-oxazole (**24**), as well as chiral ligands **L1**, **L3** and **L4** were previously disclosed in our preliminary communication.[1] The same reference contains crystallographic data for $PdCl_2$(**L1**) and $PdCl_2$(**L4**) complexes.

(1) Rubina, M.; Sherrill, W. M.; Rubin, M. *Organometallics* **2008**, *27*, 6393-6395.

Synthesis of Chiral Phosphine Ligands

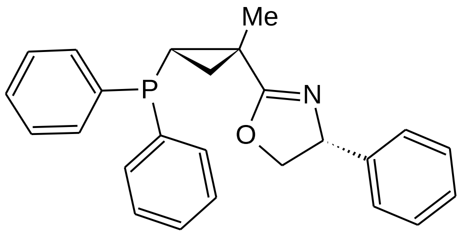

(4*R*)-2-[(1*S*,2*S*)-2-(Diphenylphosphino)-1-methylcyclopropyl]-4-phenyl-4,5-dihydro-1,3-oxazole (L2): To a stirred at −80 °C solution of (4*R*)-2-[(1*S*,2*S*)-2-bromo-1-methylcyclopropyl]-4-phenyl-4,5-dihydro-1,3-oxazole (**19**) (733 mg, 2.62 mmol) in anhydrous THF (10 mL) was added dropwise a solution of *n*-BuLi in hexane (2.5 M, 1.2 mL, 3.0 mmol). The mixture was allowed to warm up to −30 °C (within 0.5 h), after which diphenylchlorophosphine (556 μL, 684 mg, 3.1 mmol) was added dropwise, and the resulting mixture was stirred for 30 min at room temperature. The mixture was quenched with saturated aqueous solution of NH_4Cl (30 mL), and extracted with ether (3 x 15 mL). The combined ethereal phases were washed with brine, dried with $MgSO_4$ and concentrated.[2] Purification of the final product by preparative column chromatography was performed in a nitrogen-filled glove box using degassed silica gel and CH_2Cl_2/EtOAc (40:1) as an eluent. Yield 200 mg (0.52 mmol, 20%).

^{1}H NMR (400.13 MHz, C_6D_6) δ 7.72-7.68 (m, 2H), 7.67-7.63 (m, 2H), 7.29-7.15 (m, 11H), 5.15 (t, *J* = 8.3 Hz, 1H), 4.23 (dd, *J* = 10.1 Hz, 8.3 Hz, 1H), 3.86 (dd, *J* = 9.6 Hz, 8.3 Hz, 1H), 1.99 (ddd, $^2J_{PH}$ = 13.1 Hz, *J* = 7.1 Hz, 4.5 Hz, 1H), 1.62 (d, $^4J_{PH}$ = 1.5 Hz), 1.48 (ddd, $^3J_{PH}$ = 6.3 Hz, *J* = 9.1 Hz, 7.1 Hz, 1H), 0.94 (ddd, $^3J_{PH}$ = 7.3 Hz, *J* = 9.1 Hz, 4.5 Hz, 1H); ^{13}C NMR (100.67 MHz, C_6D_6) δ 169.0 (d, $^3J_{CP}$ = 3.7 Hz), 143.2, 140.8 (d, $^1J_{CP}$ = 12.5 Hz), 140.3 (d, $^1J_{CP}$ = 13.2 Hz), 133.6 (d, $^2J_{CP}$ = 19.8 Hz, +, 2C), 132.2 (d, $^2J_{CP}$ = 17.6 Hz, +, 2C), 128.8 (d, $^3J_{CP}$ = 9.5 Hz, +, 2C), 128.70 (+), 128.65 (+), 128.6 (+, 2C), 128.1 (d, $^3J_{CP}$ = 9.5 Hz, +, 2C), 127.3 (+), 127.2 (+, 2C), 74.5 (-), 70.4 (+), 26.9 (d, $^1J_{CP}$ = 12.4 Hz, +), 23.0 (d, $^3J_{CP}$ = 1.5 Hz, +), 22.3 (d, $^2J_{CP}$ = 6.6 Hz), 19.1 (d, $^2J_{CP}$ = 11.0 Hz, -); ^{31}P NMR (161.98 MHz, C_6D_6) δ -9.23; α_D^{25} -84.7^o (c 1.15, CH_2Cl_2); HRMS (TOF ES) Calculated for $C_{25}H_{24}NOPNa$ (M+Na) 408.1493, Found 408.1483 (2.5 ppm).

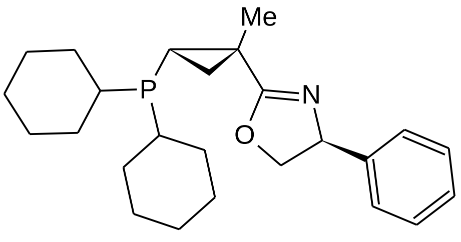

(4*S*)-2-[(1*S*,2*S*)-2-(Dicyclohexylphosphino)-1-methylcyclopropyl]-4-phenyl-4,5-dihydro-1,3-oxazole (L5): was prepared in a similar manner from 590 mg (2.10 mmol) of (4*S*)-2-[(1*S*,2*S*)-2-bromo-1-methylcyclopropyl]-4-phenyl-4,5-dihydro-1,3-oxazole (**24**) and 539 mg (2.32 mmol, 1.1 equiv) of dicyclohexylchlorophosphine. Purification of the final product by preparative column chromatography was performed in a nitrogen-filled glove box using degassed silica gel and CH_2Cl_2/EtOAc (100:1) as an eluent. Yield 228 mg (0.57 mmol, 27%).

(2) Since the material is moderately sensitive to air in solution, the work up should be performed within 10–15 min to avoid substantial oxidation.

^{1}H NMR (400.13 MHz, C_6D_6) δ 7.49 (d, *J* = 7.3 Hz, 2H), 7.31 (d, *J* = 7.3 Hz, 2H), 7.19 (d, *J* = 7.3 Hz, 1H), 5.18 (dd, *J* = 10.0 Hz, 8.3 Hz, 1H), 4.38 (dd, *J* = 10.8 Hz, 8.3 Hz, 1H), 3.93 (t, *J* = 8.3 Hz, 1H), 2.11-1.70 (m, 12H), 1.59 (s, 3H), 1.54-1.31 (m, 10H), 1.02-0.92 (m, 2H), 0.84-0.78 (m, 1H); ^{13}C NMR (100.67 MHz, C_6D_6) δ 169.6 (d, $^{3}J_{CP}$ = 2.9 Hz), 143.9, 128.6 (+, 2C), 127.44 (+), 127.40 (+), 127.3 (+), 74.6 (-), 70.5 (+), 35.1 (d, $^{1}J_{CP}$ = 13.9 Hz, +), 34.7 (d, $^{1}J_{CP}$ = 11.7 Hz, +), 31.2 (d, J_{CP} = 16.1 Hz, -), 30.9 (d, J_{CP} = 17.6 Hz, -), 29.6 (d, J_{CP} = 8.9 Hz, -), 29.5 (d, J_{CP} = 6.6 Hz, -), 27.9-27.6 (m, -, 5C), 27.0 (d, J_{CP} = 2.9 Hz, -), 23.0 (+), 22.2 (d, $^{1}J_{CP}$ = 22.0 Hz, +), 20.0 (d, $^{2}J_{CP}$ = 7.3 Hz), 18.6 (d, $^{2}J_{CP}$ = 7.3 Hz, -), ^{31}P NMR (161.98 MHz, C_6D_6) δ -4.02; α_D^{25} -116.7° (c 1.00, CH_2Cl_2); HRMS (TOF ES) Calculated for $C_{25}H_{37}NOP$ (M+H) 398.2613, Found 398.2604 (2.3 ppm).

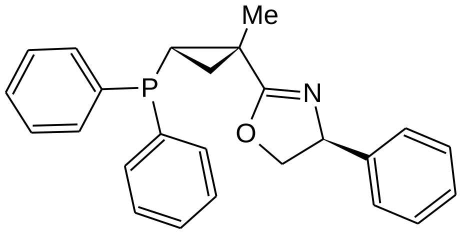

(4*S*)-2-[(1*S*,2*S*)-2-(Diphenylphosphino)-1-methylcyclopropyl]-4-phenyl-4,5-dihydro-1,3-oxazole (L6): was prepared in a similar manner from 666 mg (2.38 mmol) of (4*S*)-2-[(1*S*,2*S*)-2-bromo-1-methylcyclopropyl]-4-phenyl-4,5-dihydro-1,3-oxazole (**24**). Purification of the final product by preparative column chromatography was performed in a nitrogen-filled glove box using degassed silica gel and CH_2Cl_2/EtOAc (40:1) as an eluent. Yield 354 mg (0.92 mmol, 39%).

^{1}H NMR (400.13 MHz, C_6D_6) δ 7.73-7.64 (m, 4H), 7.44-7.42 (m, 2H), 7.31-7.15 (m, 9H), 5.07 (dd, *J* = 10.1 Hz, 8.3 Hz, 1H), 4.27 (dd, *J* = 10.1 Hz, 8.1 Hz, 1H), 3.85 (ps.-t, *J* = 8.3 Hz, 8.1 Hz, 1H), 1.98 (ddd, $^{2}J_{PH}$ = 12.9 Hz, *J* = 6.8 Hz, 4.6 Hz, 1H), 1.58 (d, $^{4}J_{PH}$ = 1.5 Hz, 3H), 1.46 (ddd, $^{3}J_{PH}$ = 6.1 Hz, *J* = 9.1 Hz, 6.8 Hz, 1H), 0.93 (ddd, $^{3}J_{PH}$ = 7.3 Hz, *J* = 9.1 Hz, 4.6 Hz, 1H); ^{13}C NMR (100.67 MHz, C_6D_6) δ 169.1 (d, $^{3}J_{CP}$ = 3.7 Hz), 143.5, 141.0 (d, $^{1}J_{CP}$ = 11.7 Hz), 140.1 (d, $^{1}J_{CP}$ = 12.4 Hz), 133.6 (d, $^{2}J_{CP}$ = 19.8 Hz, +, 2C), 132.2 (d, $^{2}J_{CP}$ = 17.6 Hz, +, 2C), 128.8 (d, $^{3}J_{CP}$ = 8.8 Hz, +, 2C), 128.7 (d, $^{3}J_{CP}$ = 10.3 Hz, +, 2C), 128.7 (+), 128.6 (+, 2C), 128.1 (+), 127.4 (+, 2C), 127.3 (+), 74.7 (-), 70.4 (+), 26.8 (d, $^{1}J_{CP}$ = 11.7 Hz, +), 22.9 (d, $^{3}J_{CP}$ = 1.5 Hz, +), 22.4 (d, $^{2}J_{CP}$ = 7.3 Hz), 18.9 (d, $^{2}J_{CP}$ = 11.0 Hz, -); ^{31}P NMR (161.98 MHz, C_6D_6) δ -9.03; α_D^{25} −178.7° (*c* 1.25, CH_2Cl_2); HRMS (TOF ES) calculated for $C_{25}H_{25}NOP$ (M+H) 386.1674, found 386.1680 (1.6 ppm).

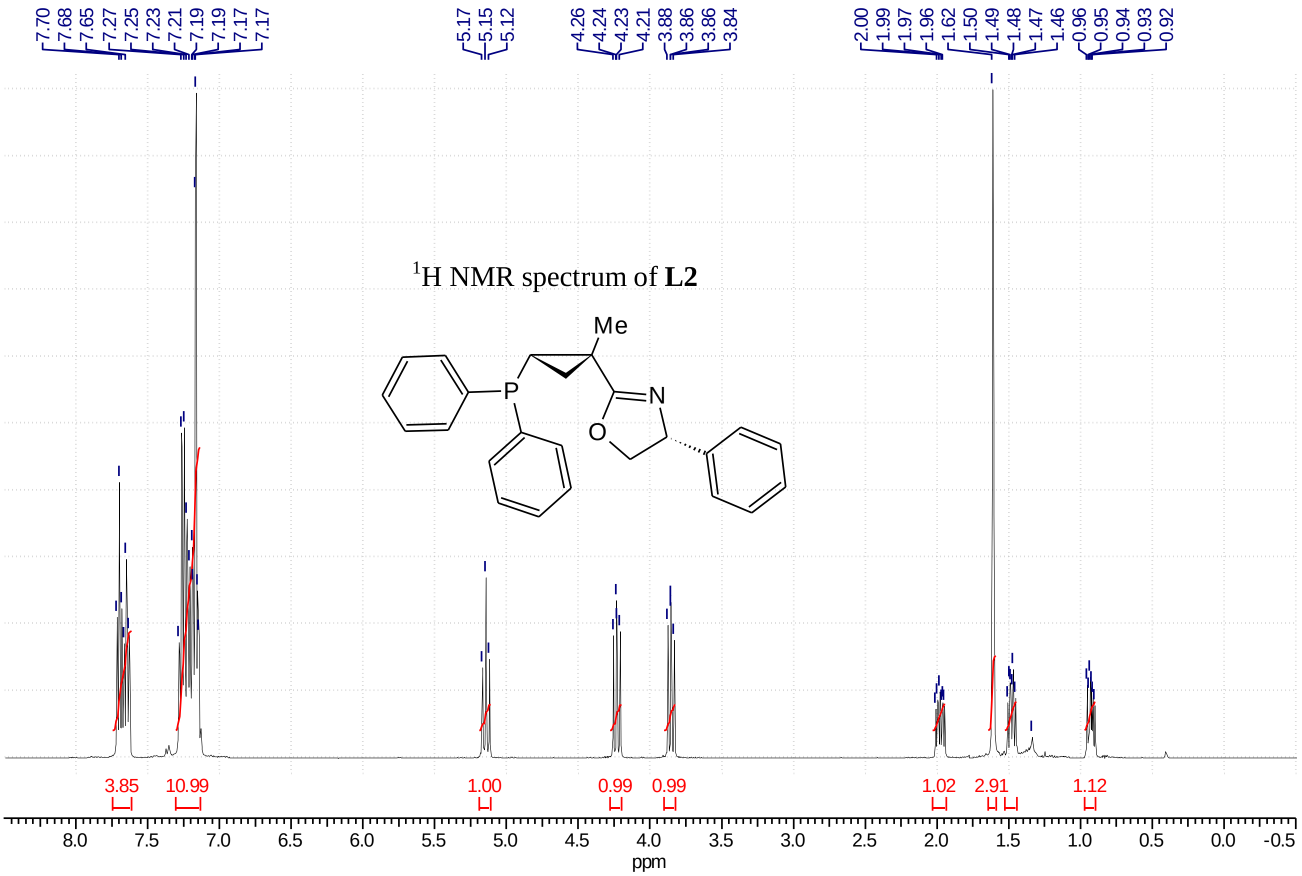

^{1}H NMR spectrum of L2
Me
P
N
O
7.70
7.68
7.65
7.27
7.25
7.23
7.21
7.19
7.19
7.17
7.17
5.17
5.15
5.12
4.26
4.24
4.23
4.21
3.88
3.86
3.86
3.84
2.00
1.99
1.97
1.96
1.62
1.50
1.49
1.48
1.47
1.46
0.96
0.95
0.94
0.93
0.92
3.85
10.99
1.00
0.99
0.99
1.02
2.91
1.12
ppm

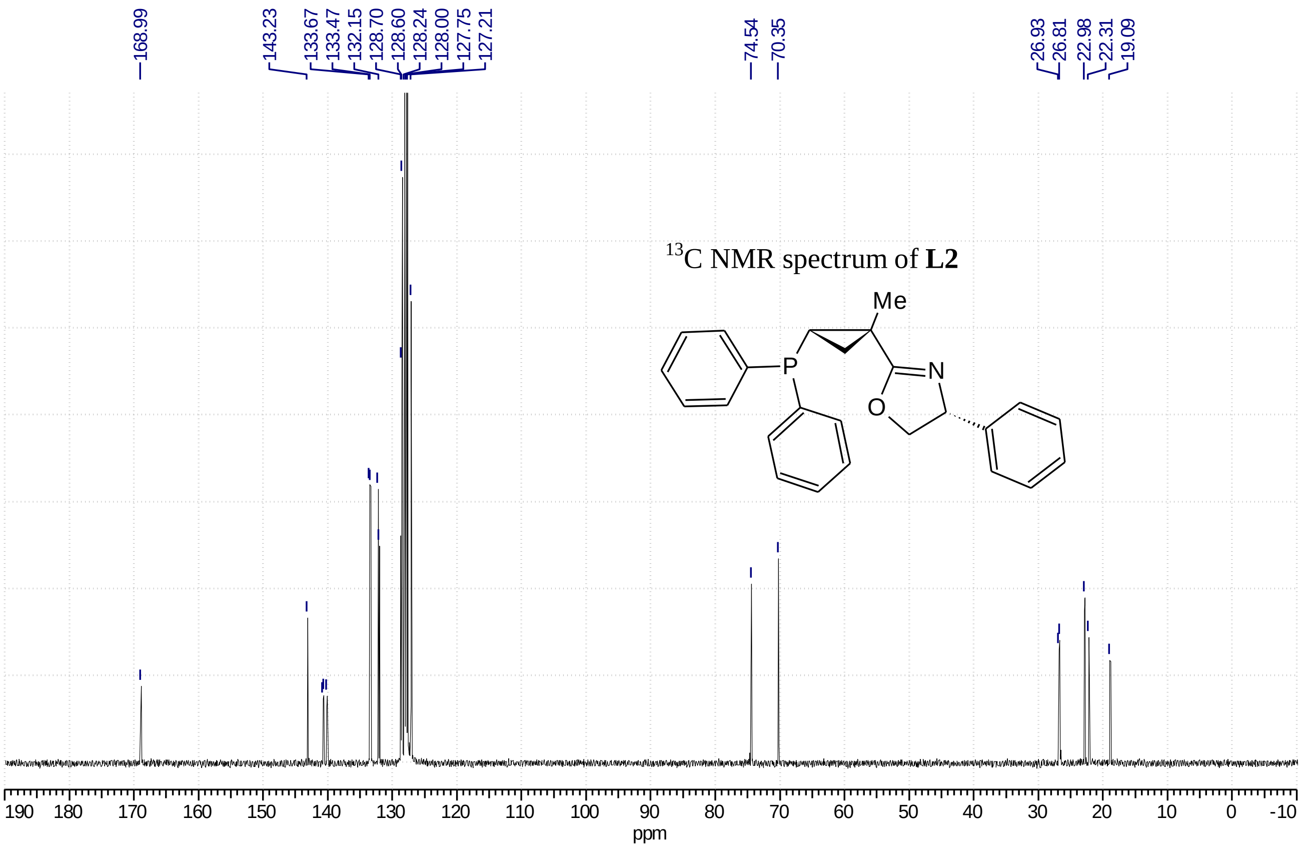

^{13}C NMR spectrum of L2
Me
P
N
O
168.99
143.23
133.67
133.47
132.15
128.70
128.60
128.24
128.00
127.75
127.21
74.54
70.35
26.93
26.81
22.98
22.31
19.09
ppm

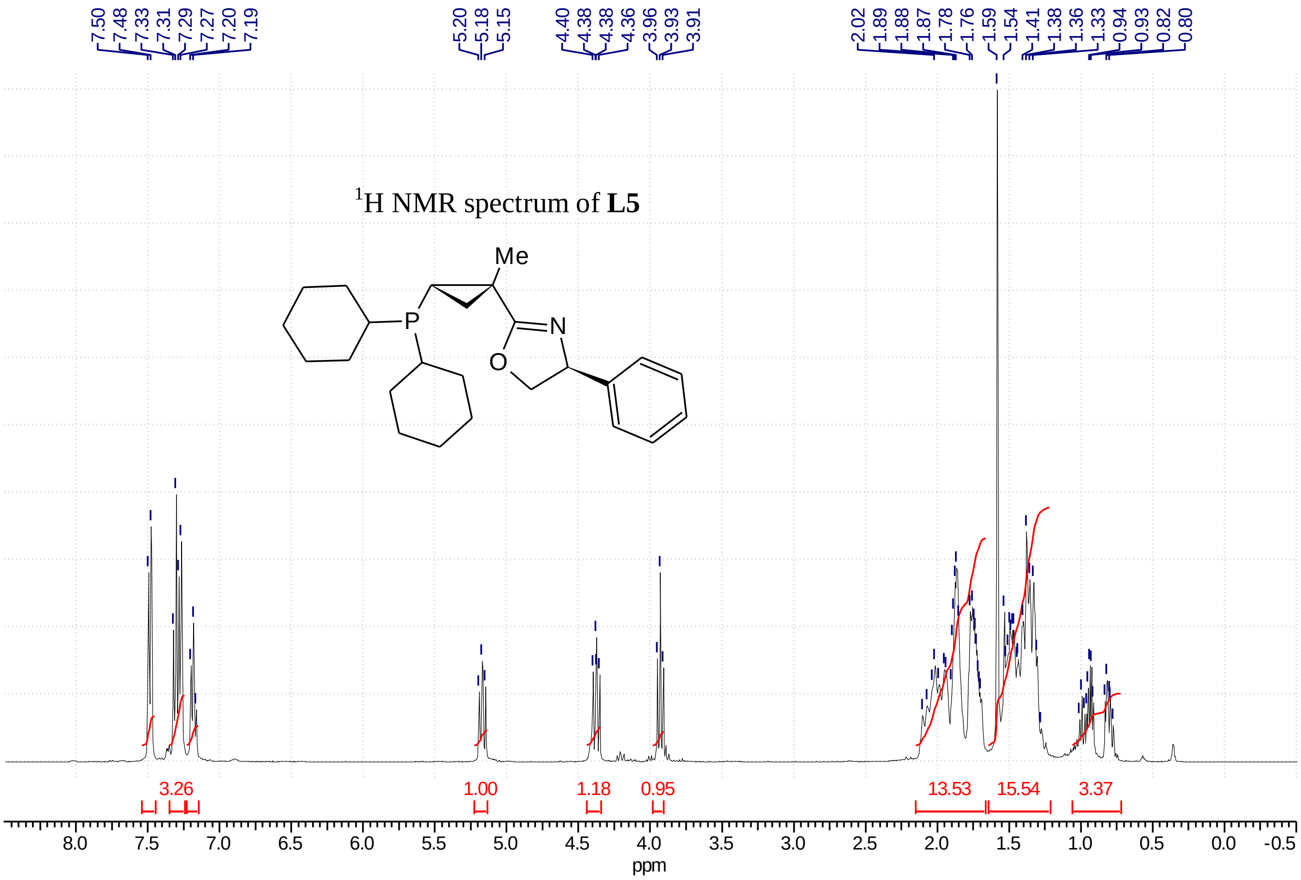
7.50 7.48 7.33 7.31 7.29 7.27 7.20 7.19
5.20 5.18 5.15
4.40 4.38 4.38 4.36 3.96 3.93 3.91
2.02 1.89 1.88 1.87 1.78 1.76 1.59 1.54 1.41 1.38 1.36 1.33 0.94 0.93 0.82 0.80
¹H NMR spectrum of L5
Me
P
N
O
3.26
1.00
1.18
0.95
13.53
15.54
3.37
ppm

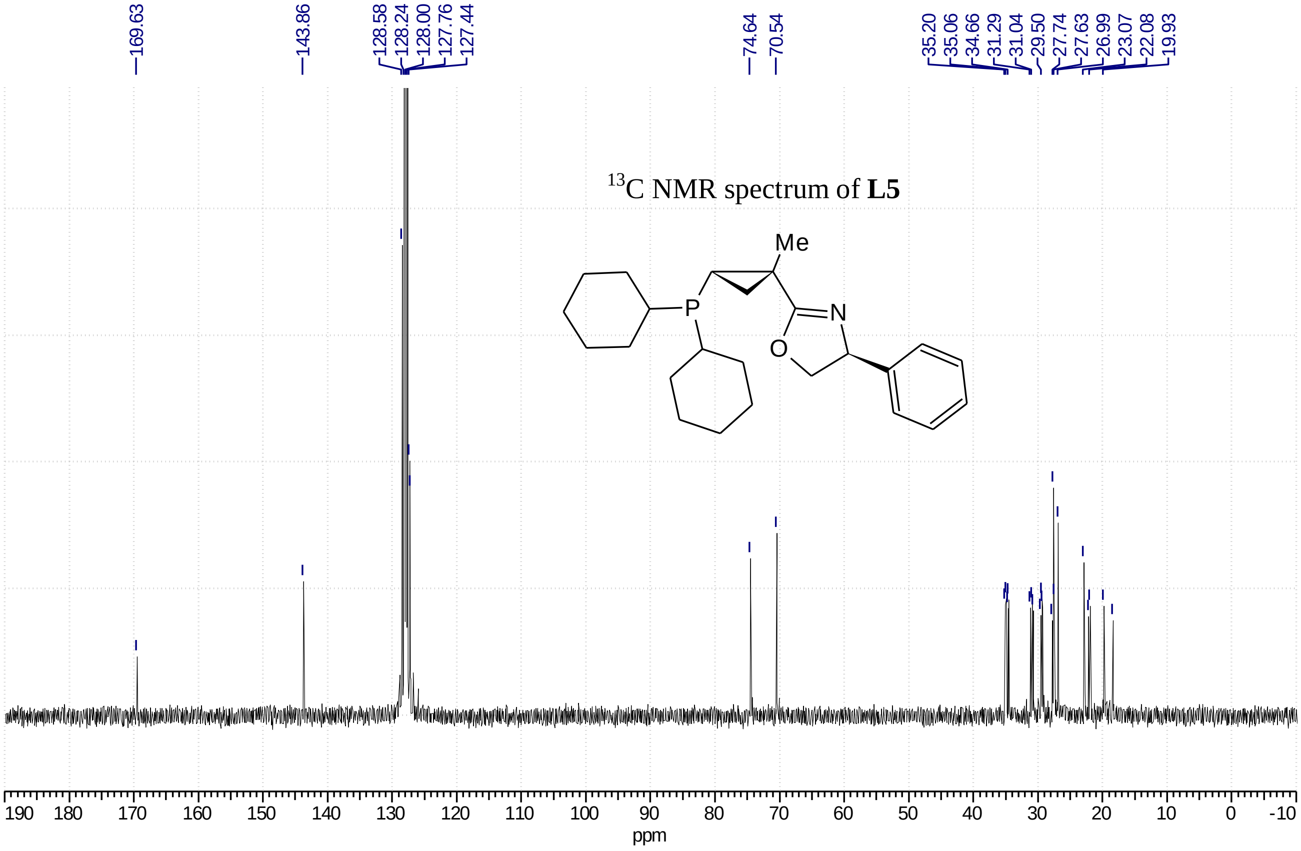
169.63
143.86
128.58 128.24 128.00 127.76 127.44
74.64 70.54
35.20 35.06 34.66 31.29 31.04 29.50 27.74 27.63 26.99 23.07 22.08 19.93
¹³C NMR spectrum of L5
Me
P
N
O
ppm

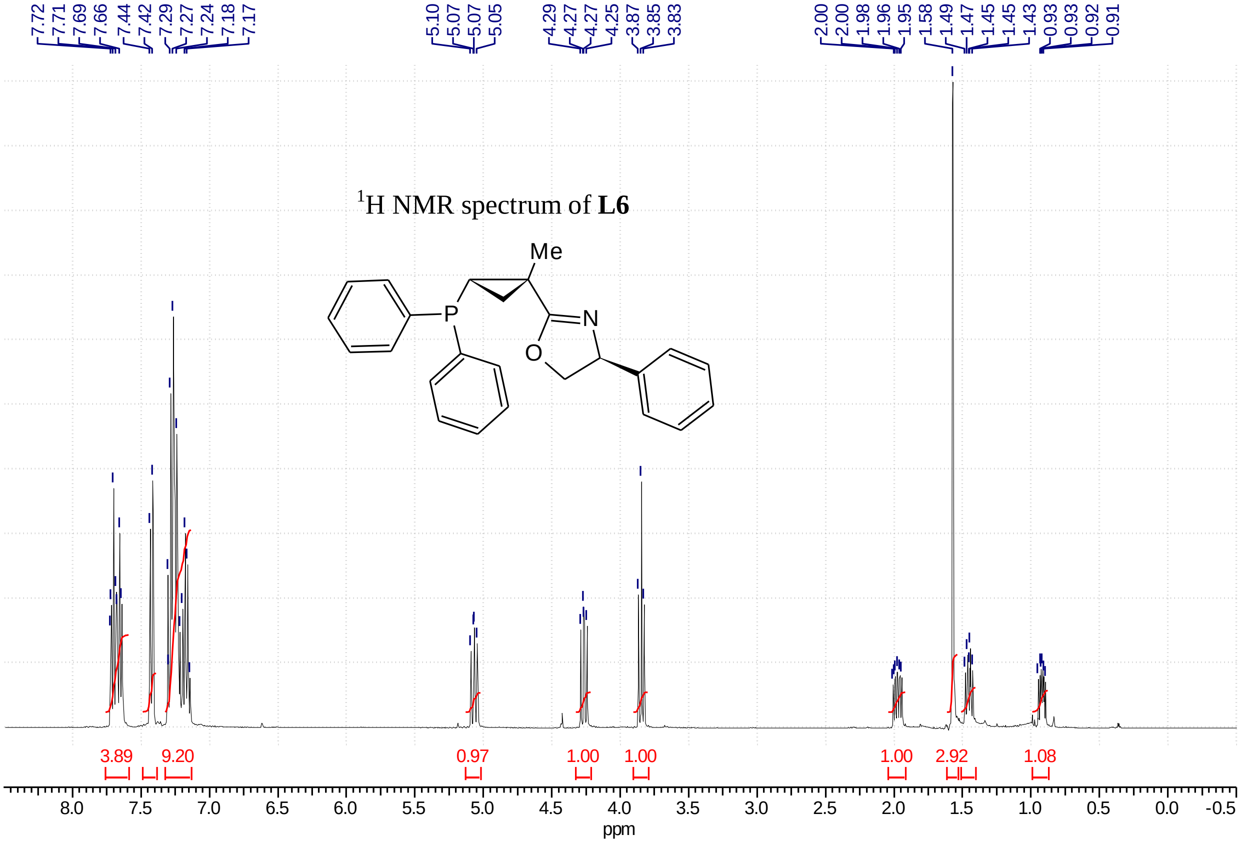

7.72 7.71 7.69 7.66 7.44 7.42 7.29 7.27 7.24 7.18 7.17
5.10 5.07 5.07 5.05
4.29 4.27 4.27 4.25 3.87 3.85 3.83
2.00 2.00 1.98 1.96 1.95 1.58 1.49 1.47 1.45 1.45 1.43 0.93 0.93 0.92 0.91
^{1}H NMR spectrum of **L6**
Me
P
N
O
3.89 9.20 0.97 1.00 1.00 1.00 2.92 1.08
8.0 7.5 7.0 6.5 6.0 5.5 5.0 4.5 4.0 3.5 3.0 2.5 2.0 1.5 1.0 0.5 0.0 -0.5
ppm

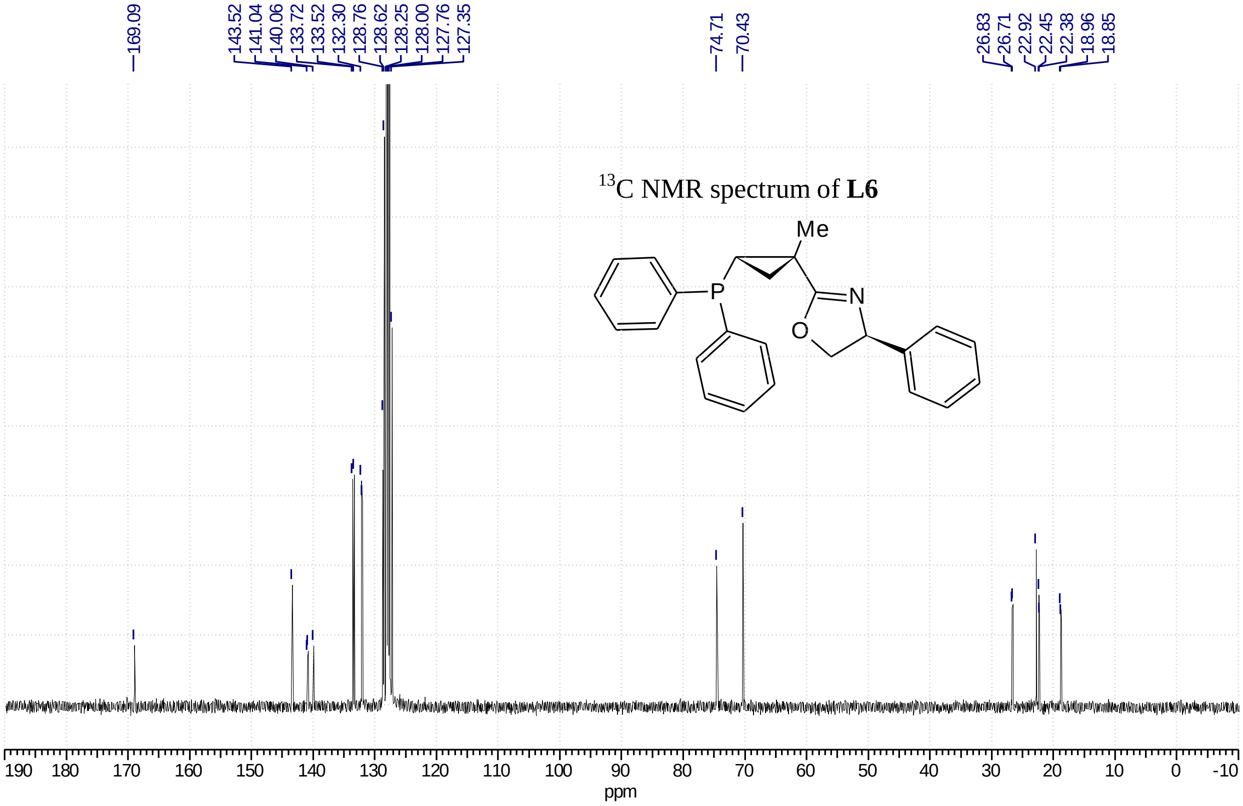

169.09
143.52 141.04 140.06 133.72 133.52 132.30 128.76 128.62 128.25 128.00 127.76 127.35
74.71 70.43
26.83 26.71 22.92 22.45 22.38 18.96 18.85
^{13}C NMR spectrum of **L6**
Me
P
N
O
190 180 170 160 150 140 130 120 110 100 90 80 70 60 50 40 30 20 10 0 -10
ppm
